# Fully-gapped superconductivity with rotational symmetry breaking in pressurized kagome metal CsV_3_Sb_5_

**DOI:** 10.1038/s41467-025-58941-w

**Published:** 2025-04-17

**Authors:** X. Y. Feng, Z. Zhao, J. Luo, Y. Z. Zhou, J. Yang, A. F. Fang, H. T. Yang, H.-J. Gao, R. Zhou, Guo-qing Zheng

**Affiliations:** 1 Institute of Physics, Chinese Academy of Sciences, and Beijing National Laboratory for Condensed Matter Physics, Beijing, 100190 China; 2 School of Physical Sciences, University of Chinese Academy of Sciences, Beijing, 100190 China; 3 School of Physics and Astronomy, Beijing Normal University, Beijing, 100875 China; 4 Key Laboratory of Multiscale Spin Physics, Ministry of Education, Beijing Normal University, Beijing, 100875 China; 5 Department of Physics, Okayama University, Okayama, 700-8530 Japan

**Keywords:** Superconducting properties and materials, Electronic properties and materials

## Abstract

The discovery of the kagome metal CsV_3_Sb_5_ has generated significant interest in its complex physical properties, particularly its superconducting behavior under different pressures, though its nature remains debated. Here, we performed low-temperature, high-pressure ^121/123^Sb nuclear quadrupole resonance (NQR) measurements to explore the superconducting pairing symmetry in CsV_3_Sb_5_. At ambient pressure, we found that the spin-lattice relaxation rate 1/*T*_1_ exhibits a kink at *T* ~ 0.4 *T*_c_ within the superconducting state and follows a *T*^3^ variation as temperature further decreases. This suggests the presence of two superconducting gaps with line nodes in the smaller one. As pressure increases beyond *P*_c_ ~ 1.85 GPa, where the charge-density wave phase is completely suppressed, 1/*T*_1_ shows no Hebel-Slichter peak just below *T*_c_, and decreases rapidly, even faster than *T*^5^, indicating that the gap is fully opened for pressures above *P*_c_. In this high pressure region, the angular dependence of the in-plane upper critical magnetic field *H*_c2_ breaks the *C*_6_ rotational symmetry. We propose the *s* + *id* pairing at *P* > *P*_c_ which explains both the 1/*T*_1_ and *H*_c2_ behaviors. Our findings indicate that CsV_3_Sb_5_ is an unconventional superconductor and its superconducting state is even more exotic at high pressures.

## Abstract (web summary)

The authors study CsV_3_Sb_5_ by nuclear quadrupole resonance. At ambient pressure, there are two superconducting gaps with line nodes in the smaller one. For pressures above *P*_c_ ~ 1.85 GPa, where the charge-density wave phase is completely suppressed, they observe fully-gapped superconductivity with broken rotational symmetry.

## Introduction

The kagome lattice, formed by a network of corner-sharing triangles, features Dirac fermions, flat bands, and van Hove singularities in its electronic structure. This makes it an ideal system for exploring geometric frustration, strongly correlated electronic states, and topological quantum phenomena^1–3^. The Dirac cones support massless charge carriers, while flat bands result in highly localized electrons through destructive interference^4^. This electron localization amplifies electron-electron interactions, potentially fostering exotic phenomena such as quantum spin liquid states^1,5,6^, fractional quantum Hall states^7,8^, and unconventional superconductivity^9,10^. While theoretical predictions suggest the unconventional pairing in kagome-lattice superconductors, most discovered materials with this structure exhibit conventional superconducting behaviors. For instance, CeRu_2_, one of the earliest superconductors with a kagome lattice^11,12^, shows an *s*-wave gap symmetry^13^. Recently, the Copper(II)-based coordination polymer Cu-BHT^14,15^ emerged as a potential unconventional superconductor, exhibiting nonexponential temperature-dependent superfluid density, although further experiments are required to confirm its superconducting gap structure.

The *A*V_3_Sb_5_ (*A* = K, Cs, Rb) family, another kagome-lattice superconductor group, has recently attracted interests due to its unique electronic properties^16–22^. At ambient pressure, CsV_3_Sb_5_ undergoes a charge density wave (CDW) transition at *T*_CDW_ = 94 K, followed by a superconducting transition at *T*_c_ = 2.5 K^23^. This CDW state is associated with unusual phenomena such as chirality^24–26^, nematicity^27^, and time-reversal symmetry breaking (TRSB)^28^. Hydrostatic pressure gradually suppresses the CDW transition, which disappears around a critical pressure, *P*_c_ ~ 1.9 GPa, while *T*_c_ forms a double-dome-shaped phase diagram, hinting at the unconventional nature of the superconductivity^29–33^. Some experimental observations indeed suggest unconventional superconducting behaviors in this system. The transport measurements reveal a two-fold rotational symmetry in the superconducting state under ambient pressure^34,35^. Other exotic features of the superconducting state, such as the presence of Majorana zero modes within vortex cores^36^, pairing density waves (PDW) and TRSB have also been observed^37–39^. However, the appearance of a Hebel-Slichter peak in NQR measurements^40^, temperature-dependent superfluid density from transverse-field muon spin rotation (*μ*SR) experiments^41,42^, and magnetic penetration depth measurements^43^ point to an *s*-wave gap. Given its nonmagnetic nature and relatively weak electron correlations, CsV_3_Sb_5_ is still often proposed to be a phonon-mediated conventional superconductor^44^, though this is still under debate. The nature of superconductivity at high pressures is also contested, partly due to a limited number of high-pressure experiments. The *μ*SR measurements suggest spontaneous TRSB just below *T*_c_ and a nodeless superconducting gap when charge order is fully suppressed^45,46^. However, NQR studies find no Hebel-Slichter peak in the spin-lattice relaxation rate 1/*T*_1_ below *T*_c_^30^, which appears inconsistent with a fully gapped state. Additional high-pressure measurements are essential to further investigate the unique properties of the superconducting state, which are crucial for understanding the pairing mechanism in CsV_3_Sb_5_.

In this study, we carried out low-temperature and high-pressure ^121/123^Sb-NQR measurements to investigate the pairing symmetry in CsV_3_Sb_5_. At ambient pressure, a distinct kink was observed in the spin-lattice relaxation rate 1/*T*_1_ at *T* ~ 0.4 *T*_c_ followed by a *T*^3^ behavior down to the lowest temperatures. This behavior indicates multiple superconducting gaps, with line nodes present in the smaller one. When the pressure exceeds *P*_c_ ~ 1.85 GPa, where the CDW phase is completely suppressed, the temperature dependence of 1/*T*_1_ indicates that the superconducting gap fully opens. Most remarkably, the angular dependence of the in-plane upper critical magnetic field, *H*_c2_, reveals an unexpected two-fold symmetry in the fully-gapped superconducting phase at *P* >  *P*_c_. We propose an *s* + *id* pairing that simultaneously explains the full gap and rotational-symmetry broken behavior of *H*_c2_. We will also discuss a possible charge redistribution below *T*_c_ in the *P* > *P*_c_ region suggested by an increase in both the NQR frequency and linewidth. Our findings provide new insights into the unconventional superconductivity of kagome metals.

## Results

### Evolution of the superconducting gap symmetry

**Fig. 1:**
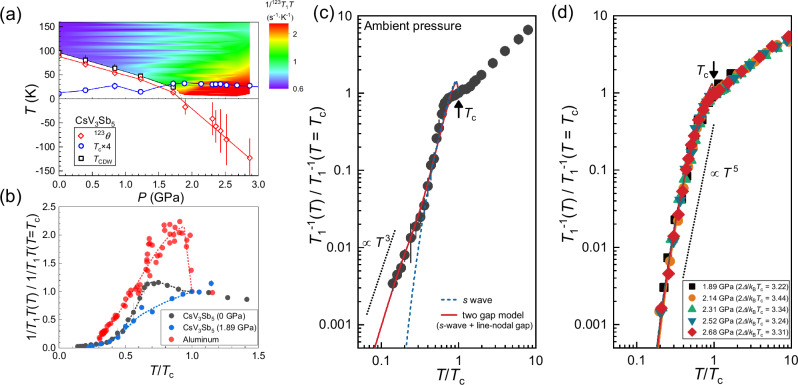
The phase diagram of CsV_3_Sb_5_ and temperature-dependent 1/*T*_1_ in the superconducting state. **a** The black squares represent *T*_CDW_ values derived from our previous NQR study^31^, while blue circles indicate *T*_c_ × 4, obtained from ac susceptibility measurements^47^. Red diamonds mark ^123^*θ*, extracted from Curie-Weiss fitting of the ^123^Sb2 NQR linewidth (see Supplementary Fig. 4(a))^47^. Color variations above *T*_CDW_ reflect the evolution of 1/*T*_1_*T* of ^123^Sb2 (see Supplementary Fig. 4(b))^47^. Solid and dashed lines are guides for the eye. The error bar for *θ* is the s.d. in the fitting of linewidth. The error bar for *T*_CDW_ reflects the temperature interval used in NQR spectra measurements^31^. The error bar for *T*_c_ corresponds to the transition width evaluated from the 10-90% criterion. **b** The black and blue dots represent temperature-dependent 1/*T*_1_*T* at ambient pressure and 1.89 GPa, respectively. The red dots represent temperature-dependent 1/*T*_1_*T* of aluminum^48^. The 1/*T*_1_*T* of aluminum shows a clear Hebel-Slichter peak below *T*_c_, while just a small Hebel-Slichter peak below *T*_c_ was observed in CsV_3_Sb_5_. Above *P*_*c*_, the Hebel-Slichter peak is absent below *T*_c_(see Supplementary Fig. 5 for *P *≥ 2.14 GPa)^47^. The dashed lines are guides for the eye. **c** Temperature dependence of the normalized spin-lattice relaxation rates [1/*T*_1_(*T*)/1/*T*_1_(*T*_c_)] at ambient pressure. The blue dashed curve represents the simulation of the *s*-wave model, while the red solid curve represents the simulation of the *s*-wave + line-nodal gap model. **d** Temperature dependence of the normalized spin-lattice relaxation rates [1/*T*_1_(*T*)/1/*T*_1_(*T*_c_)] at *P* > 1.85 GPa. Solid lines represent the simulations of the fully-gapped model with a single energy gap. The dotted line indicates *T*^3^ and *T*^5^ behaviors as visual guides in (c) and (d), respectively. The solid arrows indicate *T*_c_. The error bar in *T*_1_ is the s.d. in fitting the nuclear magnetization recovery curve.

Figure 1(a) represents the pressure-temperature phase diagram of CsV_3_Sb_5_^31^, incorporating data on the parameter ^123^*θ*, derived from fitting the NQR linewidth and spin-lattice relaxation rate 1/*T*_1_*T* within the pressure range of 2 to 3 GPa (see Supplementary Fig. 4^47^). The parameter *θ* indicates the presence of CDW correlations. As pressure *P* surpasses the critical value *P*_c_ ~ 1.85 GPa, a decrease in *θ* reflects a reduction in CDW fluctuations. At the same time, an increase in 1/*T*_1_*T* with rising pressure suggests an enhancement in spin fluctuations. In the normal state, 1/*T*_1_*T* increases only slightly with decreasing temperature at *P* < *P*_*c*_, but is enhanced rapidly when temperature decreases at *P* > *P*_*c*_. To explore the superconducting gap symmetry under varying pressures, we measured the temperature dependence of 1/*T*_1_ in the superconducting state. Figure 1(b) displays the temperature dependence of 1/*T*_1_*T* for CsV_3_Sb_5_. At ambient pressure, we also observe a small Hebel-Slichter coherent peak below *T*_c_, consistent with previous NQR studies^30,40^. The presence of this coherence peak typically suggests the *s*-wave symmetry in the superconducting gap. However, its magnitude is notably smaller than that observed in conventional superconductors such as aluminum (see Fig. 1(b))^48^. As temperature decreases, 1/*T*_1_ drops sharply below *T*_c_ but changes to a *T*^3^ dependence below *T* ~ 0.4 *T*_c_ as shown in Fig. 1(c). The *T*^3^ variation is a characteristic behavior of line nodes in the gap function^49,50^. The relaxation rate 1/*T*_1_ below *T*_c_ is expressed as^51^

1 $$\frac{{T}_{1}({T}_{{{\rm{c}}}})}{{T}_{1s}}=\frac{2}{{k}_{B}{T}_{c}}\int\left(1+\frac{{\Delta }^{2}}{{E}^{2}}\right){N}_{s}{(E)}^{2}f(E)[1-f(E)]dE$$

where ${N}_{s}(E)={N}_{0}E/\sqrt{{E}^{2}-{\Delta }^{2}}$ is the density of state (DOS) in the superconducting state, Δ is the magnitude of the energy gap, *f*(*E*) is the Fermi distribution function, and the (1 + Δ^2^/*E*^2^) is the coherence factor. For an *s*-wave gap, the coherence factor and the divergence of the DOS at *E* = Δ will lead to a Hebel-Slichter peak just below *T*_c_^52,53^. First, we used a single *s*-wave gap model to simulate 1/*T*_1_, with Δ_*s*_ = 1.85*k*_B_*T*_c_, represented by the blue dashed line in Fig. 1(c). However, we found that the simulation results at low temperatures did not align with the experimental results. Considering that there exists a *T*^3^ temperature-dependent relationship at low temperatures, we then used a two gap model, namely an *s*-wave gap  + a line-nodal gap($\Delta (\Phi )={\Delta }_{0}\cos (2\Phi )$), to simulate the 1/*T*_1_ results.

We assume that the total superconducting DOS is contributed from two gaps as *N*_tot_ = *α* ⋅ *N*_*s*_ + (1 − *α*) ⋅ *N*_*L*_, where *N*_*s*_ and *N*_*L*_ are the superconducting DOS from the *s*-wave and line-nodal gap, respectively. Using such two-gap model allowed us to successfully fit the data over the entire temperature range shown in Fig. 1(c), yielding Δ_*s*_ = 2.0*k*_B_*T*_c_ and Δ_*L*_ = 0.63*k*_B_*T*_c_ with a ratio of DOS on two bands (*N*_*s*_: *N*_*L*_ = 0.9: 0.1). These results suggest that CsV_3_Sb_5_ features a small superconducting gap with line nodes. Nevertheless, both the relative weight and the gap size of the fully-opened gap are found to be much larger than this nodal gap. We also note that theoretical calculations on the kagome lattice have indicated the possibility of unconventional pairing states with a sign-changing gap structure, particularly *d*-wave gap, which may manifest as a small Hebel-Slichter peak in the temperature-dependent 1/*T*_1_ due to the destructive sublattice interference effects^54^. It is probable that these factors result in a small coherence peak in 1/*T*_1_ as shown in Fig. 1(c). Given that both the size and the weight of the nodal gap are extremely small compared to the fully opened gap, the signature of this gap can only be observed at very low temperatures, which is indeed in agreement with the observation of a residual DOS in scanning tunneling spectroscopy^37,38^. This is precisely why it was challenging to detect previously. In any case, our results show that CsV_3_Sb_5_ is already an unconventional superconductor at ambient pressure. The small component of the line-nodal gap is consistent with the temperature dependence of 1/*T*_1_*T* in the normal state which points to weak spin fluctuations (see Fig. 1(a)).

Next, we examine the 1/*T*_1_ results at high pressures. For pressures above *P*_c_ ~ 1.85 GPa, we observe a rapid decrease in 1/*T*_1_ without a Hebel-Slichter coherence peak just below *T*_c_ (see Fig. 1(b) and Supplementary Fig. 5). At low temperatures, the decrease of 1/*T*_1_ is very steep, even faster than the *T*^5^-variation (see Fig. 1(d) and Supplementary Fig. 6), indicating a full gap. Such behavior is similar to the strong coupling superconductor Ca_3_Ir_4_Sn_13_^55^ and iron-based superconductor Ba_0.68_K_0.32_Fe_2_As_2_^56^, where the Hebel-Slichter peak is absent in the fully gapped superconducting state. Nevertheless, the simulations of 1/*T*_1_ indicate that the gap size of CsV_3_Sb_5_ (2Δ ~ 3.3 *k*_B_*T*_c_) is significantly smaller than that of Ca_3_Ir_4_Sn_13_ (2Δ = 4.42 *k*_B_*T*_c_). Hence, it is unlikely that the absence of the Hebel-Slichter coherence peak of CsV_3_Sb_5_ is attributed to the strong coupling. Instead, it might be similar to that of iron-based superconductors, which could be related to the sign-reversed gap structure of the superconducting gap^57^ and the strong spin fluctuations^58^, and this is in line with our observation of the enhanced spin fluctuations above *P*_c_ in CsV_3_Sb_5_(see Fig. 1(a)). To further explore the structure of the superconducting gap, we measured the in-plane upper critical magnetic field *H*_c2_ as shown below.

### Two-fold symmetry of *H*_c2_

**Fig. 2:**
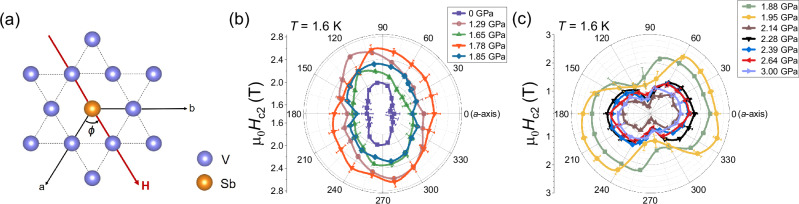
Evidence for persistent two-fold symmetry of *H*_c2_ at high pressures. **a** The illustration depicts the field orientation in the hexagonal plane with respect to the *a* axis (angle *ϕ*), where an angle of *ϕ* = 0^∘^ corresponds to *H*∥*a* axis. **b**, **c** represent the angular dependence of *H*_c2_ respectively below and above *P*_c_. The data are obtained from ac susceptibility measurements at a fixed temperature *T* = 1.6 K (see Supplementary Fig. 7)^47^. The error was estimated from uncertainties in the determination of *H*_c2_ due to scatter in the raw field sweeps.

By rotating the sample with the magnetic field applied in the *ab* plane, we measured the angular dependence of *H*_c2_ under pressures at *T* ~ 1.6 K (see Supplementary Fig. 7 for the field dependence of the ac susceptibility at various pressures^47^). Here, *ϕ* represents the angle between the in-plane field orientation and the *a*-axis, as illustrated in Fig. 2(a). The angular dependence of *H*_c2_ at different pressures, shown in Fig. 2(b) and (c), reveals a clear two-fold symmetry at all pressures. However, the symmetry of the *H*_c2_ changes across *P*_c_. At *P *≤ 1.85 GPa, the maximum *H*_c2_ value occurs when field direction is nearly perpendicular to the *a*-axis, indicating the maximum superconducting gap alignment in this direction. By contrast, when the pressure is larger than 1.85 GPa, the field direction corresponding to the maximum *H*_c2_ becomes approximately perpendicular to that at lower pressures, as shown in the Fig. 2(b) and (c). Namely the *H*_c2_ symmetry changes by 90^∘^.

When the applied pressure is smaller than *P*_c_, the two-fold symmetry of superconductivity can be linked to the presence of the CDW order. Firstly, the nematicity in the CDW state can induce an anisotropic Fermi surface^27,35^, and then leads to a two-fold symmetry of the *H*_c2_. Secondly, superconductivity emerges in the coexist state of the CDW order and loop current state, which can also have a two-fold symmetry of *H*_c2_^59^. Besides these, the two-fold anisotropy in *H*_c2_ could also stem from the nodal superconducting gap component indicated by the *T*^3^ behavior. We note that the phase of the *H*_c2_ symmetry reported from the *c*-axis resistivity^35^ and specific heat^60^ measurements differs from each other and also from our results. This suggests that the *H*_c2_ symmetry for *P* < *P*_c_ is not due to intrinsic pairing symmetry, but is attributable to the crystal distortion or disorders in the sample. The crystal distortion or disorders can affect the electronic nematicity or loop current order, and thus affect the phase of the *H*_c2_ symmetry. Obviously, more detailed experimental investigations are still required to disclose the physical nature of the two-fold symmetry of *H*_c2_ for *P* < *P*_c_.

However, with the increasing pressure above *P*_c_, where the CDW order is completely suppressed and CDW fluctuations weaken, we observe a two-fold *H*_c2_ in which the direction of the maximum *H*_c2_ changed by nearly 90^∘^ (see Fig. 2(c)). Such an observation at high pressures is unpredicted. It is important to note that the observed two-fold symmetry of superconductivity in the *P* > *P*_c_ region cannot be ascribed to the CDW order. Moreover, no other rotational-symmetry breaking state has been reported in the normal state for *P* > *P*_c_, so it is also difficult to attribute the two-fold symmetry in *H*_c2_ to a Fermi surface anisotropy. Therefore, the two-fold symmetry of *H*_c2_ is related to the intrinsic superconducting pairing symmetry. In the carrier-doped Bi_2_Se_3_ superconductors^61–64^, the two-fold symmetry is due to a pinning of the *d*-vector of the spin-triplet superconductivity^61,65^. However, there is no evidence of spin-triplet in CsV_3_Sb_5_. Considering that the superconducting gap is fully opened and the time-reversal symmetry was found to be broken by high-pressure *μ*SR measurements^46^, we propose a scenario in which a two-component superconductivity of *s* + *id* pairing appears for *P* > *P*_c_. Such state was previously proposed in non-trivial multiband superconductors such as iron-based superconductors^66,67^ and heavy-fermion superconductors^68,69^. This pairing state violate the time-reversal symmetry and also the *C*_4_ rotation symmetry^66^. Although the *s*-wave component might have six-fold symmetry due to the *D*_6*h*_ point group symmetry of CsV_3_Sb_5_ as observed by specific heat measurements^60^, the *d*-wave component of the *s* + *id* state will result in a two-fold symmetry of *H*_c2_. Meanwhile, the fully-opened superconducting gap of *s* + *id* pairing naturally explains the temperature dependence of 1/*T*_1_ below *T*_c_.

### Electric field gradient anomaly

**Fig. 3:**
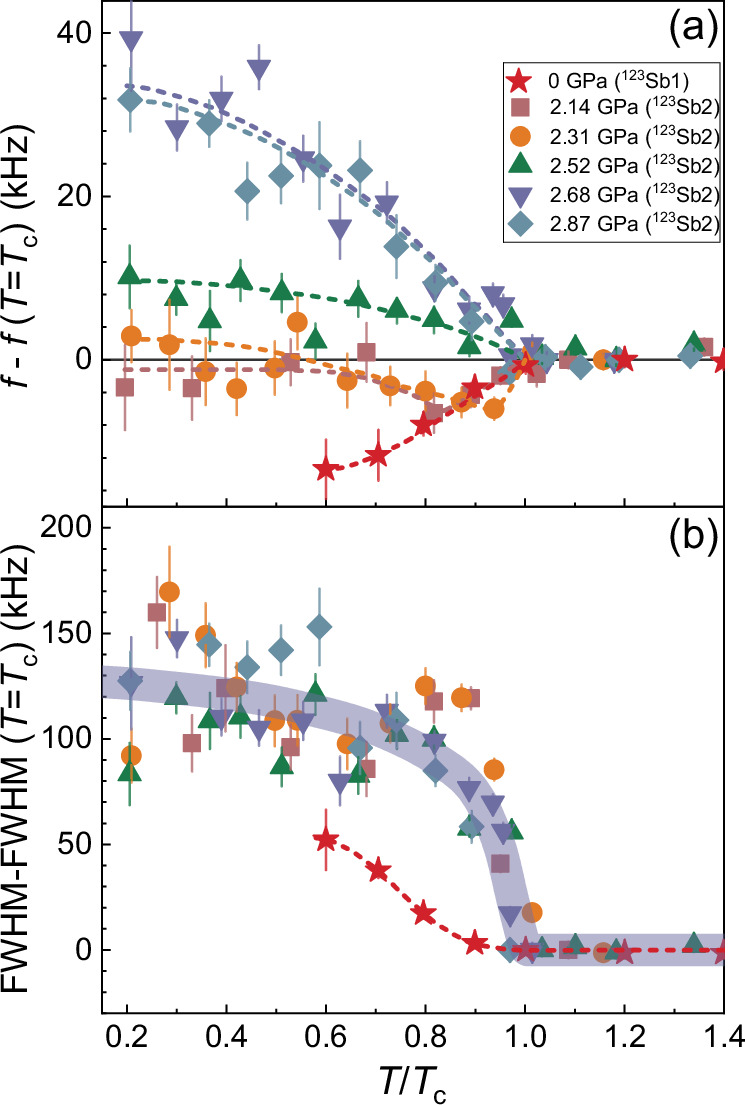
NQR frequency and linewidth in the superconducting state. **a** The temperature dependence of ^123^Sb1 (ambient pressure) and ^123^Sb2 (*P *≥ 2.14 GPa) NQR frequency *f* after subtracting the *f*(*T* = *T*_c_)^47^. **b** The temperature dependence of the full width at half maximum (FWHM) of ^123^Sb1 (ambient pressure) and ^123^Sb2 (*P *≥ 2.14 GPa) NQR spectra after subtracting FWHM(*T* = *T*_c_) under different pressures. The solid and dashed lines are guides for the eyes. Error bars are s.d. in the fits of the NQR spectra.

Figure 3(a) shows the temperature dependence of the NQR frequency at various pressures. At ambient pressure, a reduction in the frequency is observed below *T*_c_ as presented in Fig. 3(a). A similar anomaly in the temperature dependence of NQR frequency was also observed in YBa_2_Cu_4_O_8_^70^, and it is attributed to lattice anomalies resulting from the strong electron-phonon coupling of the electrons. Specifically, the thermal expansion coefficient in CsV_3_Sb_5_ significantly increases along the *c*-axis below *T*_c_^71^, indicating the further *c*-axis lattice distortions in the superconducting state.

**Fig. 4:**
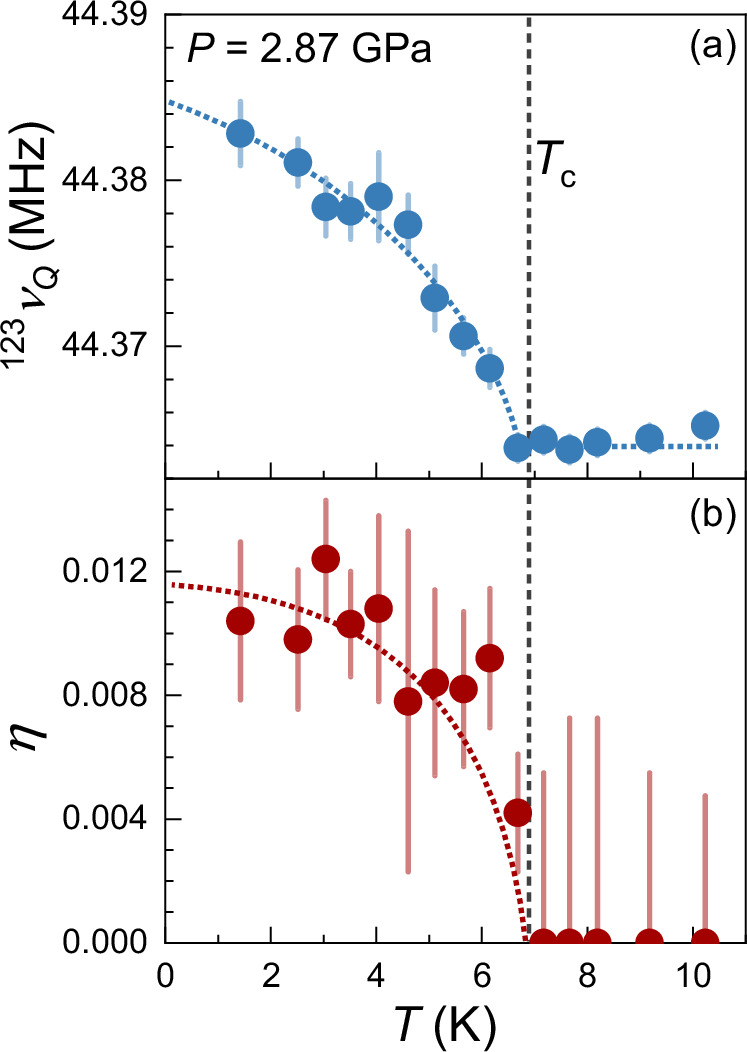
The obtained *ν*_*Q*_ and *η* in the superconducting state. **a**, **b** are respectively the temperature dependence of *ν*_*Q*_ and asymmetry parameter *η* of ^123^Sb2 at *P* = 2.87 GPa, which are deduced from the ^121^Sb2 and ^123^Sb2 NQR spectra^47^. The dashed line indicates *T*_c_. Error bars are s.d. in the fits of the NQR spectra.

At *P* > *P*_c_ region, instead of the decrease of the NQR frequency, an increase of the NQR frequency is observed below *T*_c_. For *P* = 2.14 and 2.31 GPa, the NQR line initially shifts to the lower frequency below *T*_c_, but begins to increase below *T* ~ 0.9 *T*_c_, and eventually a significant enhancement can be seen at *P* > 2.5 GPa. For *P* > *P*_c_ region, the opposite shift of the NQR line compared to the ambient pressure suggests that the main factors causing the observed shift of the NQR lines are different. Due to a more substantial shift in ^121/123^Sb-NQR lines in the superconducting state^47^, we can obtain the precise temperature dependence of *ν*_*Q*_ and *η* for *P* = 2.87 GPa, as shown in Fig. 4. Below *T*_c_, not only *ν*_*Q*_, but also *η* increases. The linewidth also increases as the temperature decreases (see Fig. 3(b)), indicating the emergence of the distribution of both *ν*_*Q*_ and *η*. One possible explanation for all these behaviors might be a charge redistribution resulting from the composite pairing in the superconducting state, as predicted in some heavy fermion superconductors by previous theoretical works^72–74^. In materials with mixed valence, it has been proposed that the composite pairing emerges as a low-energy consequence of valence fluctuations in two distinct symmetry channels. This kind of pairing can lead to a mixing of empty and doubly occupied states, causing a redistribution of charge associated with the superconducting transition.

As a result, this charge redistribution is anticipated to induce changes in the EFG around nuclear sites and shifts in NQR frequency below *T*_c_. However, there is no evidence indicating that CsV_3_Sb_5_ is a mixed-valent metal. We also note that localized magnetic moments were suggested to play a direct role in pairing involving the condensation of bound states with conducting electrons in heavy fermion superconductors. Although we observed the enhancement of spin fluctuations under high pressures, there is no evidence of the formation of local moments in CsV_3_Sb_5_. Therefore, more works, both theoretical and experimental, are needed in this aspect.

Another possible explanation is more directly associated with the *s* + *id* pairing state. A previous theoretical study has shown that the local *C*_4_ lattice rotation symmetry would be broken due to the spontaneous currents near disorders in *s* + *id* superconducting states^66^. This should lead to an increase of *η* as we observed in Fig. 4(b). In this scenario^66^, disorders in the sample would naturally lead to a distribution of both *η* and *ν*_*Q*_, and result in the quadrupole broadening of the NQR lines in the superconducting state as we found (Fig. 3(b))^47^.

## Discussions

We move to discuss the properties and pairing symmetry change across *P*_c_. For *P* < *P*_c_, we observed that the 1/*T*_1_ follows a *T*^3^ behavior below *T* < 0.4 *T*_c_ at ambient pressure, which indicates the line nodes in the superconducting gap. Nevertheless, both the relative weight and the gap size of this nodal gap are found to be significantly smaller than the fully opened gap. Recent theoretical study proposed that there can be possible accidental nodes along the *c*-axis for *P* < *P*_c_ due to the spin fluctuations^75^. For this type of pairing, the existence of nodes within the superconducting gap will not affect the symmetry of *H*_c2_, which is in accordance with the observation of different phase of the *H*_c2_ symmetry in different studies^35,60^.

For *P* > *P*_c_, we note that the spin fluctuations are prominently enhanced after the CDW is suppressed (see the phase diagram in Fig. 1(a)), which was not taken into account in previous theoretical studies. Generally, spin fluctuations can help form the *d*-wave pairing as observed in cuprates^49^. At ambient pressure where spin fluctuations are weak, the size of the nodal gap is small and its contribution to superconductivity is also relatively small. With the increasing pressure, the enhancement of spin fluctuations leads to an enhancement of *d*-wave. This may cause the *s*-wave and *d*-wave gaps to become degenerate, then would facilitate the *s* + *id* pairing. Meanwhile, the enhancement of the *d*-wave component also leads to the reduction of the Hebel-Slichter peak as we observed (see Fig. 1(b)). The *s* + *id* state is less well studied. Our findings can inspire more microscopic experimental and theoretical studies.

## Summary

In conclusion, we performed low-temperature and high-pressure ^121/123^Sb-NQR measurements to investigate the superconducting pairing symmetry in CsV_3_Sb_5_. At ambient pressure, we observed a distinct kink in 1/*T*_1_ at *T* ~ 0.4 *T*_c_, which then follows a *T*^3^ behavior down to the lowest temperatures. This behavior indicates multiple superconducting gaps, with line nodes present in the smaller one. When the pressure exceeds *P*_c_ ~ 1.85 GPa, where the CDW phase is completely suppressed, 1/*T*_1_ decreases faster than a *T*^5^ dependence below *T*_c_. Although no Hebel-Slichter peak was observed, our data indicate that the superconducting gap fully opens for pressures above *P*_c_. Most remarkably, the angular dependence of the in-plane upper critical magnetic field *H*_c2_ shows an unexpected two-fold symmetry in this high-pressure and fully-gapped superconducting phase. We propose the multi-component *s* + *id* pairing to coherently explain the results. An increase in both the NQR frequency and linewidth also occurs in the *P* > *P*_c_ region, which may also be understood as arising from a breaking of the local *C*_6_ lattice rotational symmetry due to the *s* + *id* pairing. Our results indicate that the CsV_3_Sb_5_ is an unconventional superconductor and its superconducting state is even more exotic at *P* > *P*_c_, which provides new insights into the unconventional superconducting properties of kagome metals.

## Methods

### Sample preparation and NQR measurement

High-quality single crystals of CsV_3_Sb_5_ were synthesized through the self-flux method^76^. The size of the single crystal used for *H*_c2_ measurements is approximately 2 mm × 2 mm × 0.1 mm. A commercial BeCu/NiCrAl clamp cell from Beijing Easymaterials Technology Co,.Ltd was employed as the pressure cell, and Daphne oil 7373 was utilized as a transmitting medium^77^. The CsV_3_Sb_5_ single crystal flakes were placed inside the pressure cell along with Cu_2_O powder, which is used for pressure calibration^78^ via its ^63^*ν*_*Q*_ NQR frequency (refer to Supplementary Fig. 1 for the ^63^Cu NQR spectra of Cu_2_O under different pressures)^47^. The solidification of the pressure medium Daphne 7373 occurs at *P* > 2.2 GPa at room temperature^77^. When pressurizing the pressure cell, we heat it up to at least 320 K to prevent the pressure medium Daphne 7373 from solidifying, ensuring a hydrostatic environment. NQR measurements were conducted using a phase-coherent pulsed NQR spectrometer. ^121/123^Sb spectra were obtained by sweeping the frequency point by point and integrating the spin-echo signal. The 1/*T*_1_ was determined using the saturation-recovery method. At ambient pressure, measurements below *T* = 1.5 K were conducted using a ^3^He-^4^He dilution refrigerator. To test the heat-up effect induced by RF pulses in the superconducting state, we adopt the same method as that of Pustogow et al^79^. During the experiment in the superconducting state, we utilized less than one-third of the highest available energy we found. Further details can be found in Supplementary Fig. 3^47^.

### *H*_c2_ measurements

The single crystal used for the ac susceptibility measurements has naturally formed edges with the angle of about 120^∘^ for neighboured edges^35^, which allows us to determine the crystallographic axes. The pressure cell was mounted onto the probe of a large bore cryostat such that the *ab* plane of the crystal was parallel to the magnetic field. The ac susceptibility was measured by the inductance of an *in* *situ* NQR coil. For the ac susceptibility measurement, Cu_2_O is not used for pressure calibration to preclude the influence on the measurement. The applied pressure was obtained by the value of Sb2 NQR frequency at *T* = 100 K^31^. Angle-dependent measurements were carried out by rotating the pressure cell with a custom-made rotator. The accuracy in the in-plane angle *ϕ* is around a few degrees. The *H*_c2_ was extracted from the measured ac susceptibility, which is defined as a point off the straight line drawn from high-field value (the normal state)^47^. One may argue that the two-fold symmetry at high pressures can be induced by the misalignment of the field direction to the *c*-axis. Indeed, although we cannot avoid this misalignment, however, this is unlikely for our results. All measurements under various pressures were conducted on the same single crystal through a continuous sequence of pressurizing and depressurizing cycles, without any reassembly of the pressure cell. The misalignment of the sample can not explain the nearly 90^∘^ change of the direction of the maximum *H*_c2_ across *P*_c_.

## Supplementary information

Supplementary Information

## Source data

Transparent Peer Review file

Source Data

## Data Availability

Any additional data that support the findings of this study are available from the corresponding author upon request. Source data are provided with this paper.
