# Supplementary material for: Fully-gapped superconductivity with rotational symmetry breaking in pressurized kagome metal CsV3Sb5
Source: Nat Commun. 2025 Apr 17;16:3643. doi: 10.1038/s41467-025-58941-w (PMC12003820; doi:10.1038/s41467-025-58941-w)
Supplement: Supplementary file 1 — Supplementary Information [file 41467_2025_58941_MOESM1_ESM.pdf]

Supplementary Materials for Fully-gapped superconductivity with rotational symmetry breaking in pressurized kagome metal CsV_3Sb_5

X. Y. Feng,[1,2] Z. Zhao,[1,2] J. Luo,[1] Y. Z. Zhou,[1,2] J. Yang,[1] A. F. Fang,[3,4] H. T. Yang,[1,2] H.-J. Gao,[1,2] R. Zhou,[1,2,*] and Guo-qing Zheng[5]

[1]*Institute of Physics, Chinese Academy of Sciences, and Beijing National Laboratory for Condensed Matter Physics,Beijing 100190, China*

[2]*School of Physical Sciences, University of Chinese Academy of Sciences, Beijing 100190, China*

[3]*School of Physics and Astronomy, Beijing Normal University, Beijing 100875, China*

[4]*Key Laboratory of Multiscale Spin Physics, Ministry of Education, Beijing Normal University, Beijing 100875, China*

[5]*Department of Physics, Okayama University, Okayama 700-8530, Japan*

(Dated: April 7, 2025)

*Electronic address: rzhou@iphy.ac.cn

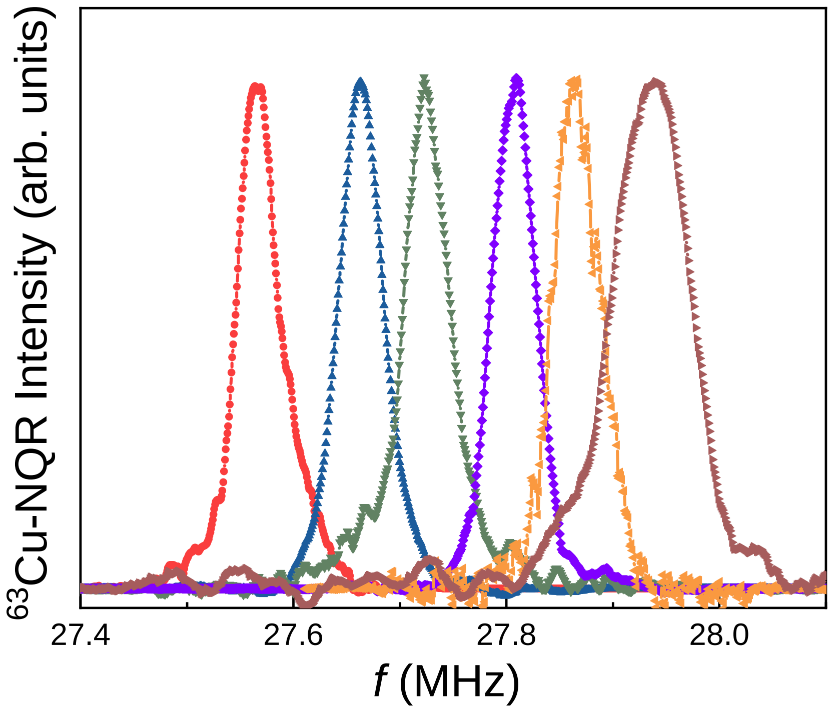

Supplementary Figure 1: ^{63}Cu NQR spectra of Cu_2O in the coil at $T \sim 5$ K for 1.89 GPa $\leq P \leq$ 2.87 GPa. The deduced pressure values by fitting a formula in a previous NQR study[1] are shown in Supplementary Table 1.

TABLE S1: ^{63}Cu NQR frequency f at $T \sim 5$ K and the corresponding pressure values for 1.89 GPa $\leq P \leq$ 2.87 GPa.

f (MHz)	27.567	27.663	27.725	27.807	27.865	27.936
P (GPa)	1.89	2.14	2.31	2.52	2.68	2.87

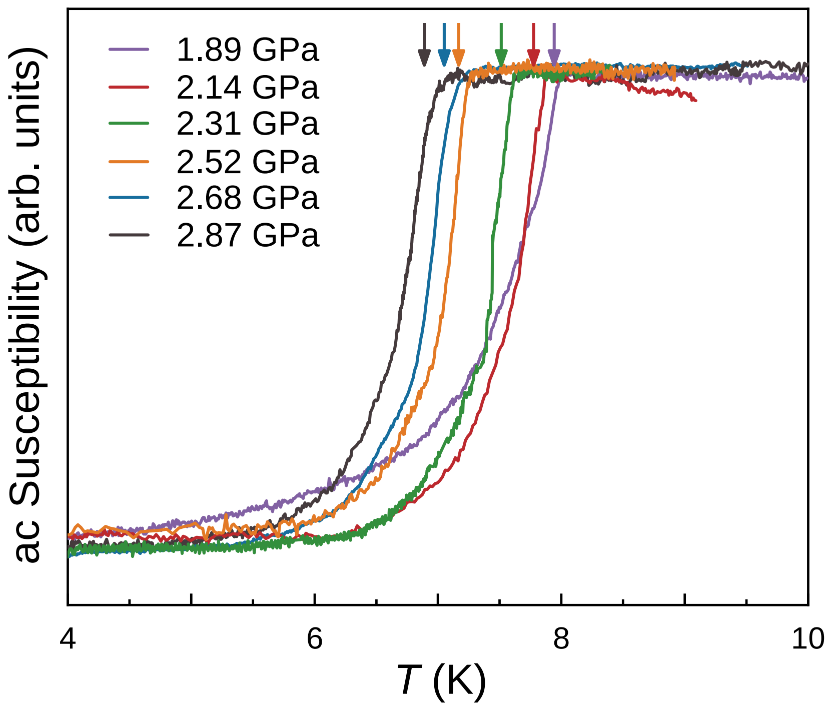

Supplementary Figure 2: The temperature dependence of the ac susceptibility measured by using an in-situ NQR coil at various pressures from 1.89 GPa to 2.87 GPa. Solid arrows represent the superconducting transition temperature T_c.

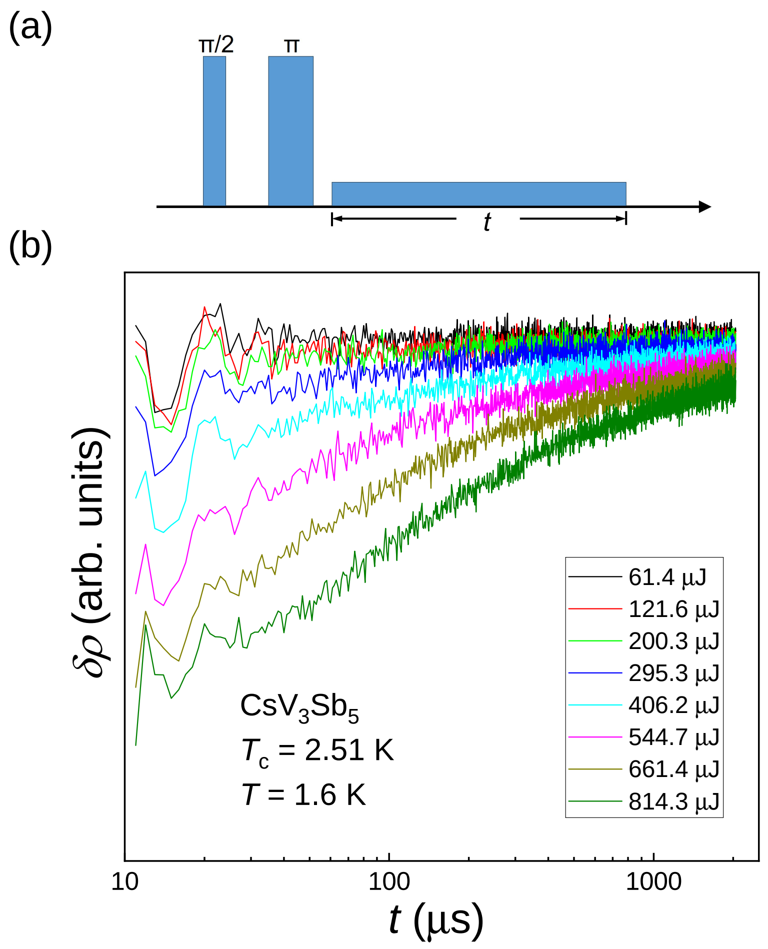

Supplementary Figure 3: (a) The illustration of the pulse sequence to examine the heat-up effect. After the standard $\pi/2-\pi$ sequence, a continuous wave pulse with a duration of t was applied. The NMR receiver then monitored the time-dependent reflected power ($\delta\rho$) from the tank circuit. (b) The time-dependent reflected power $\delta\rho$ with different energy powers at $T = 1.6$ K which is below its $T_c = 2.51$ K. As shown in Fig. S3(b), for a small energy less than 200.3 μJ, the reflected power $\delta\rho$ is independent of t. For larger energy values, the $\delta\rho$ increases with increasing t, indicating a heating effect. In our experiment, data were collected through the utilization of RF pulses with a power three times smaller than 200 μJ.

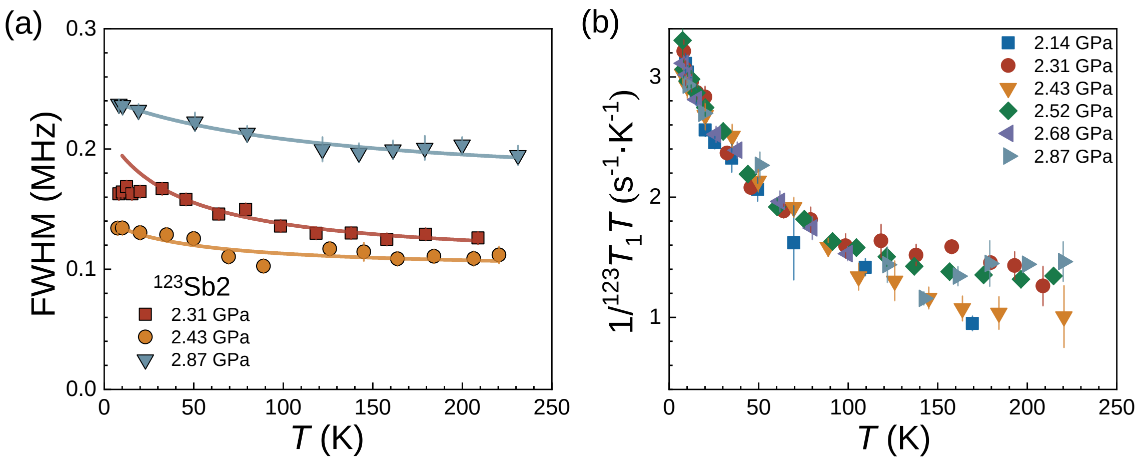

Supplementary Figure 4: Temperature dependence of the ^{123}Sb2 NQR linewidth(a) and $1/T_1T$(b) within the pressure range from 2.14 to 2.87 GPa. The solid lines are Curie-Weiss fits, and the obtained $^{123}\theta$ values together with the $1/T_1T$ were plotted in the phase diagram of CsV_3Sb_5 (see Fig. 1(a)). More details about the Curie-Weiss fitting can be found in Ref. [2]. The error bar in linewidth is s.d. in the fits of the NQR spectra. The error bar in $1/T_1T$ is the s.d. in fitting the nuclear magnetization recovery curve.

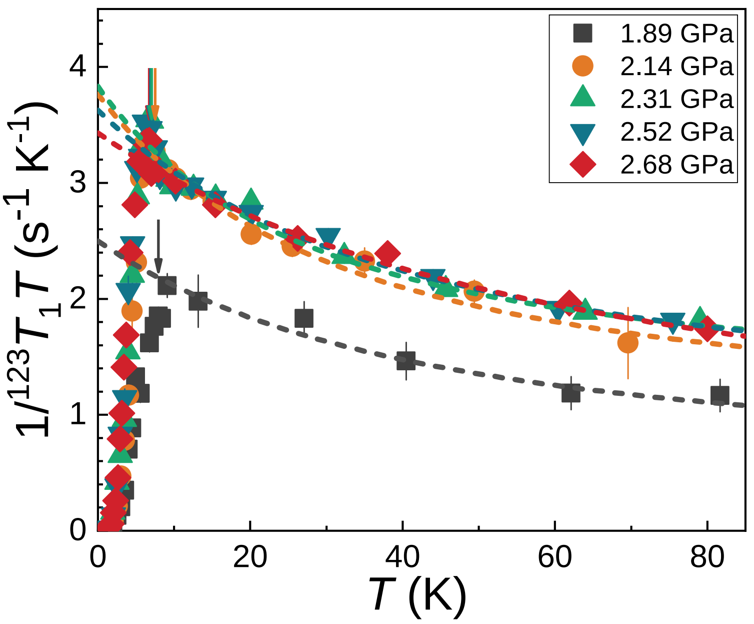

Supplementary Figure 5: The temperature-dependent $1/T_1T$ of CsV_3Sb_5 within the pressure range of 1.89 GPa $\leq P \leq$ 2.68 GPa. The dashed curves represent spin fluctuations under various pressures. The solid arrows indicate T_c. The error bars are from the s. d. in fitting the nuclear magnetization recovery curve.

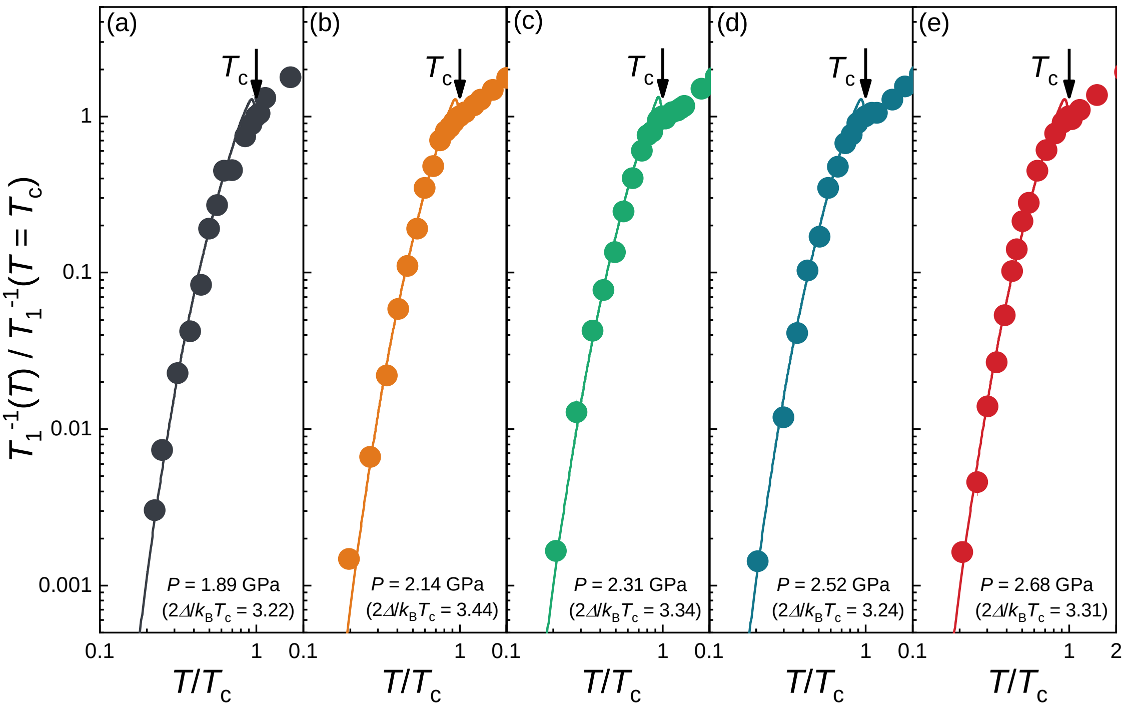

Supplementary Figure 6: Temperature dependence of the normalized spin-lattice relaxation rates $[1/T_1(T)/1/T_1(T_c)]$ for 1.89 GPa $\leq P \leq$ 2.68 GPa. The solid lines represent simulations based on the fully-gapped model with a single energy gap. The solid arrows indicate T_c. The error bars in $1/T_1$ correspond to the s.d. in fitting the nuclear magnetization recovery curve.

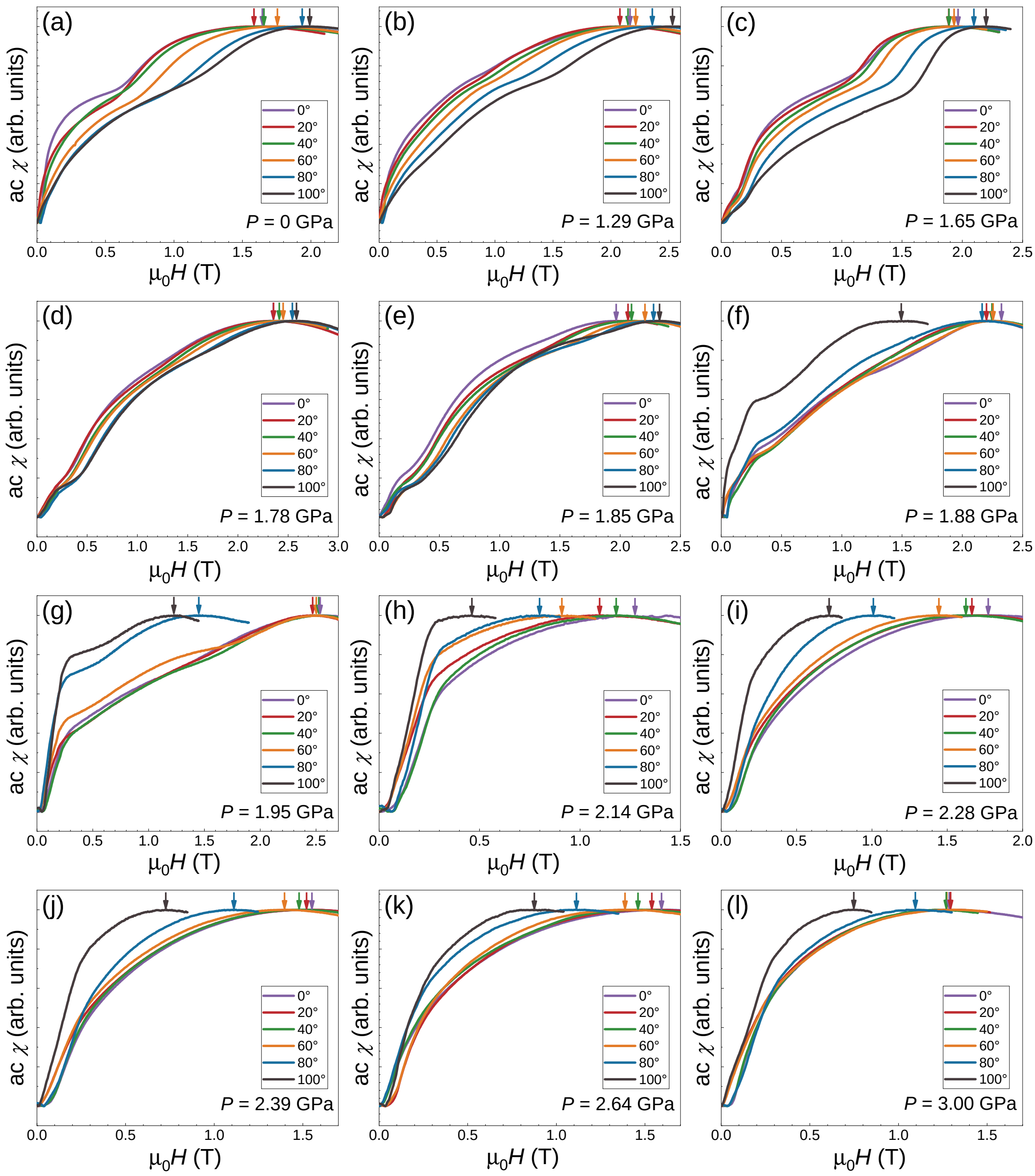

Supplementary Figure 7: Magnetic field dependence of the ac susceptibility at different in-plane angles at various pressures. The arrows indicate H_{c2} for each angle, which is defined as a point off the straight line drawn from high-field value (the normal state). For small fields, a small increase of the diamagnetic response is visible for some pressures and forms kinks in the field dependence of the ac susceptibility. This might be associated with the vortex motion, which was also observed in Sr_2RuO_4[3]. Since these occur under a magnetic field much lower than H_{c2}, they will not affect the determination of the H_{c2}.

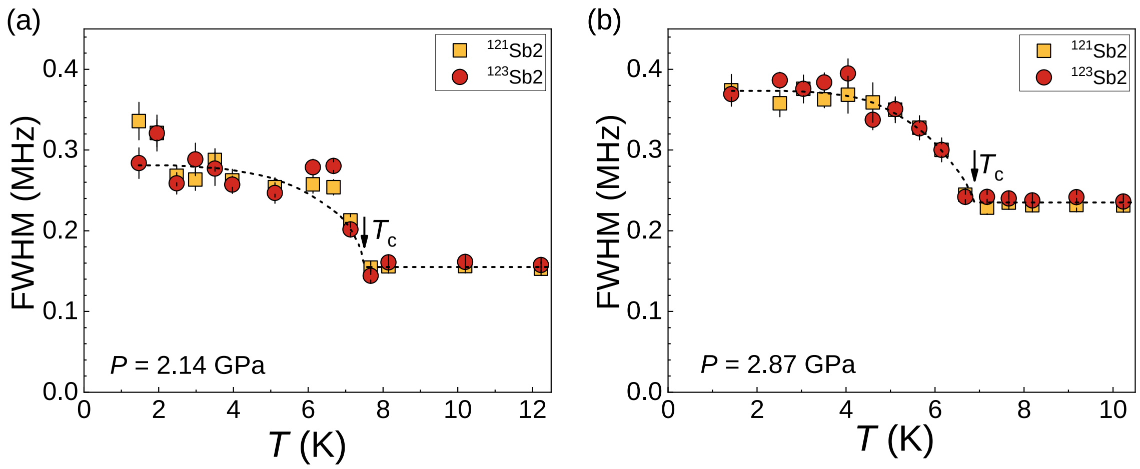

Supplementary Figure 8: The temperature dependence of $^{121/123}$Sb2 NQR linewidth at 2.14 GPa and 2.87 GPa. The NQR linewidth is ascribed to quadrupole broadening and magnetic broadening. If quadrupole broadening predominates, the ratio of the linewidths for ^{121}Sb and ^{123}Sb nuclei is associated with the ratio of their quadrupole frequencies, which is 1.18. If the magnetic broadening prevails, this ratio is anticipated to correspond to the ratio of their gyromagnetic ratios, which is 1.85. Our observation shows that the ratio of the linewidths for ^{121}Sb and ^{123}Sb nuclei is $\sim$ 1.0, indicating that quadrupole broadening is the dominant factor contributing to the NQR linewidth in the superconducting state. Given that the error bars in the line width of the Sb site are $\sim$ 10 kHz, our measurements are unable to detect an internal field less than 1 mT at the Sb sites. It is noted that the internal field obtained from the μSR experiment was merely 0.04 mT[4]. Although the internal field could be significantly different at the muon and the Sb sites, the internal field at the Sb sites might still be extremely small and undetectable in our NQR measurements. The error bar in linewidth is s.d. in the fits of the NQR spectra.

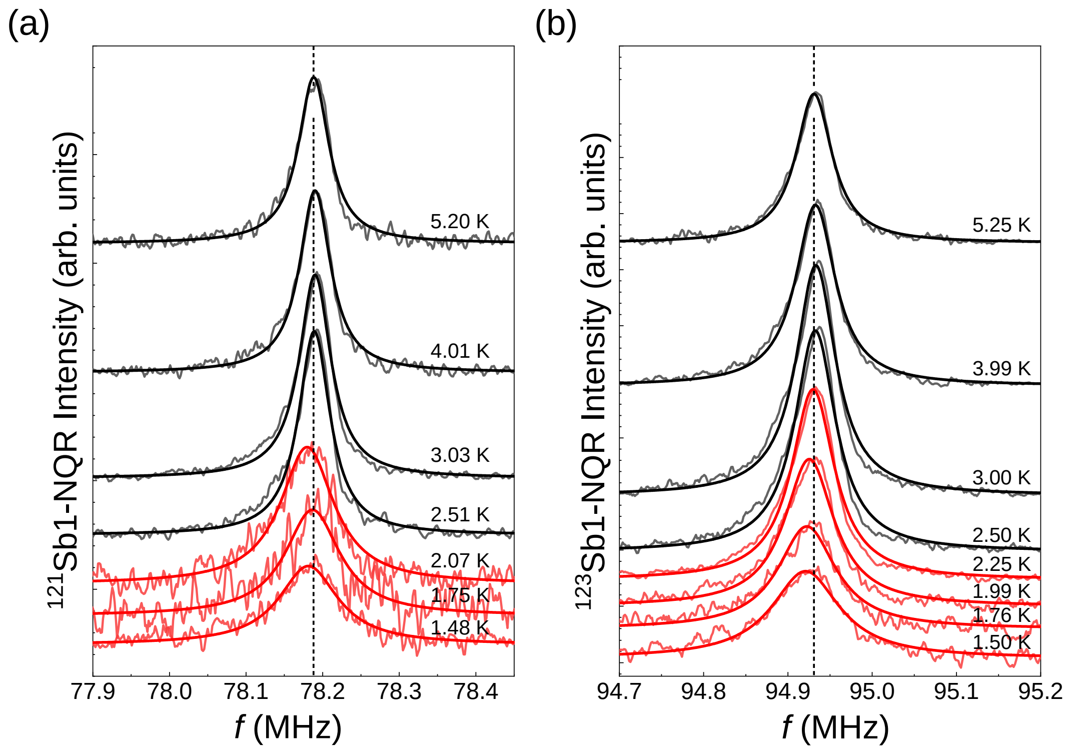

Supplementary Figure 9: The temperature dependence of ^{121}Sb1(a) and ^{123}Sb1(b) NQR spectra at ambient pressure. The black peaks represent the spectra in the normal state, while the red peaks represent the spectra in the superconducting state. The solid lines are the fits by the Lorentz function. The dashed lines are the guides for the eye.

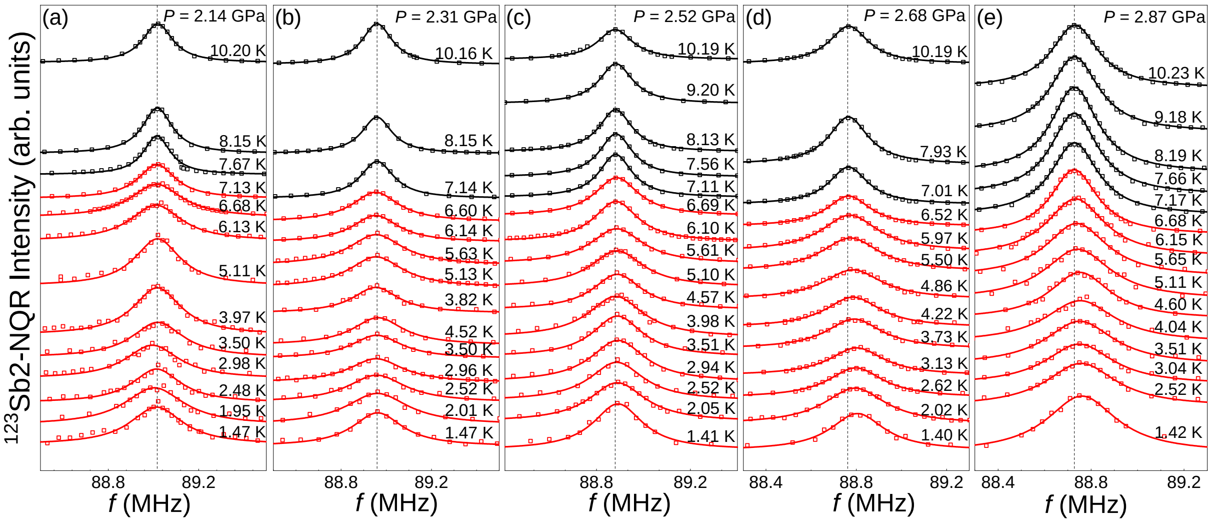

Supplementary Figure 10: The temperature dependence of ^{123}Sb2 NQR spectra at various pressures. The black peaks represent the spectra in the normal state, while the red peaks represent the spectra in the superconducting state. The solid lines are the fits by the Lorentz function. The dashed lines are the guides for the eye.

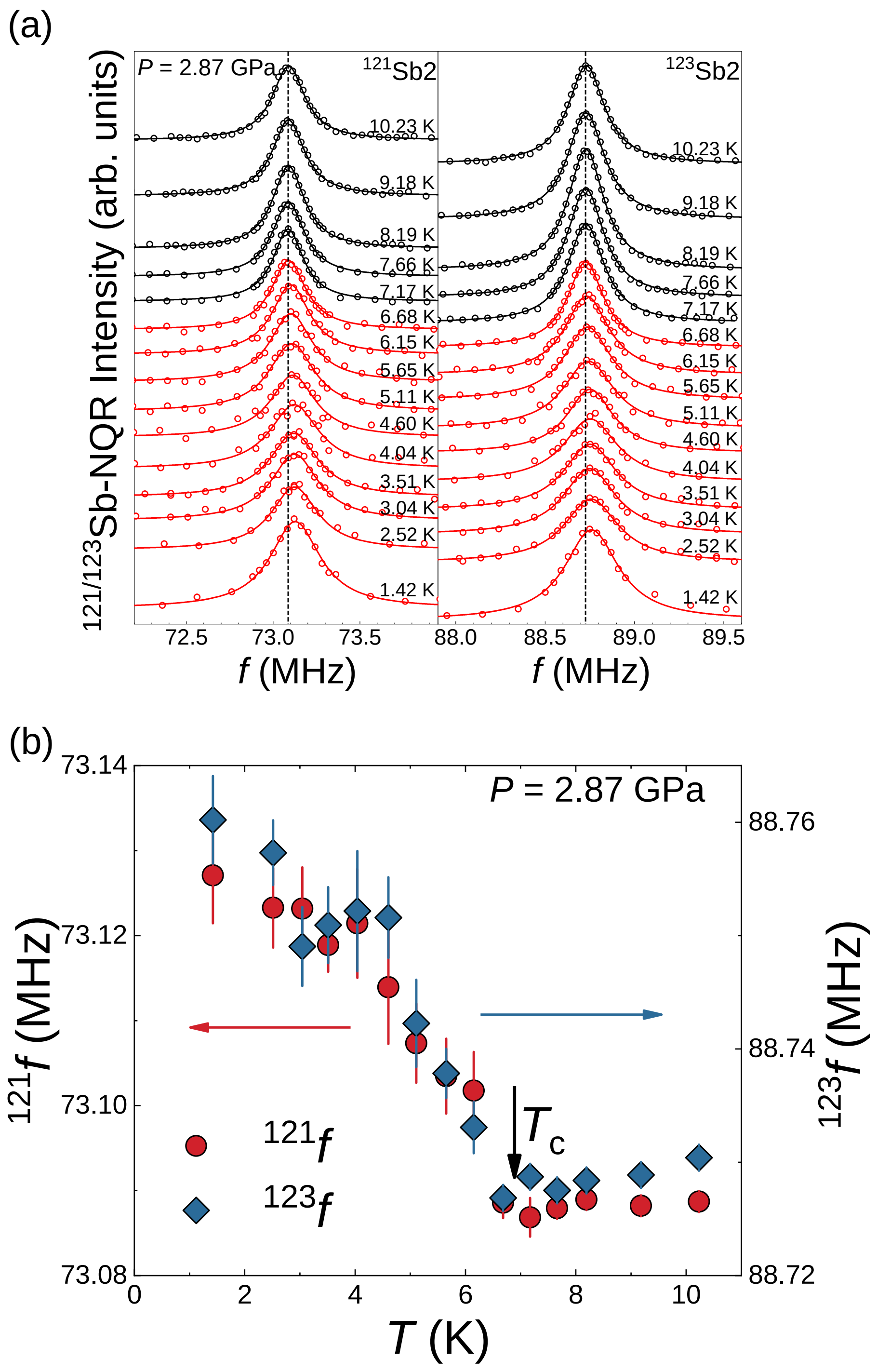

Supplementary Figure 11: (a) Temperature dependence of $^{121/123}$Sb2 NQR spectra at P = 2.87 GPa. The black peaks represent the spectra in the normal state, while the red peaks represent the spectra in the superconducting state. The solid lines are the fits by the Lorentz function. The dashed lines are the guides for the eye. (b) The temperature dependence of the ^{121}Sb2 (red circles) and ^{123}Sb2 (blue diamonds) NQR frequencies at P = 2.87 GPa. The error bars are s.d. in the fits of the NQR spectra. Since the quadrupole moments of the two Sb isotopes $^{121/123}$Sb are known, both ν_q and η can be extracted from the ^{121}Sb2 and ^{123}Sb2 lines as shown in (a).

[1] Kitagawa, K. et al. Space Efficient Opposed-Anvil High-Pressure Cell and Its Application to Optical and NMR Measurements up to 9 GPa. *J. Phys. Soc. Jpn.* **79**, 024001 (2010).

[2] Feng, X. Y. et al. Commensurate-to-incommensurate transition of charge-density-wave order and a possible quantum critical point in pressurized kagome metal CsV_3Sb_5. *npj Quantum Mater.* **8**, 23 (2023).

[3] Jerzembeck, F. et al. Upper critical field of Sr_2RuO_4 under in-plane uniaxial pressure. *Phys. Rev. B* **107**, 064509 (2023).

[4] Gupta, R. et al. Two types of charge order with distinct interplay with superconductivity in the kagome material CsV_3Sb_5. *Commun. Phys.* **5**, 232 (2022).
